# Impact of type and level of stabilizers and fermentation period on the nutritional, microbiological, and sensory properties of short‐set Yoghurt

**DOI:** 10.1002/fsn3.2507

**Published:** 2021-08-04

**Authors:** Chinazom Martina Eze, Kehinde Oludayo Aremu, Emmanuel Oladeji Alamu, Thomas Muoneme Okonkwo

**Affiliations:** ^1^ Department of Food Science and Technology University of Nigeria Nsukka Enugu State Nigeria; ^2^ Food and Nutrition Sciences Laboratory International Institute of Tropical Agriculture (IITA), Southern Africa Hub Chelstone Lusaka Zambia

**Keywords:** carboxyl methylcellulose, fermentation time, short set yoghurt, stabilizers, yoghurt properties

## Abstract

This study aimed to produce short set yoghurt with different stabilizers at different concentrations and determine the effects of the stabilizers and length of fermentation on the nutritional, microbiological, and sensory properties of short set yoghurt. Stabilized yoghurt samples were produced using 0%, 0.5%, and 1.0% concentrations of carboxyl methylcellulose (CMC), corn starch, and gum acacia with different fermentation periods (1–5 hr), respectively. Samples were analyzed for the proximate, physicochemical, microbial, and sensory properties using standard laboratory methods. Results showed that an increase in stabilizer concentration and fermentation time decreased the moisture content but increased the total solids, protein, fat, ash, sugars, pH level, and total titratable acidity. The viscosity of the yoghurt samples significantly (*p* < .05) increased with the addition of stabilizers (1.48 ± 0.03 cP to 275.57 ± 4.08 cP), with CMC having the highest increase (*p* < .05) and gum acacia the least. However, the lactic acid production reduced as the concentration of stabilizers increased but showed an increase with fermentation time. The total viable count (TVC) reduced significantly (*p* < .05) with an increase in the concentration of stabilizer and fermentation time. Hence, short set yoghurt samples containing CMC yielded highest protein (0.5%), fat (1.0%), and ash contents (1.0%). Yoghurt samples produced with a 1.0% concentration of gum acacia gave an optimum pH (0.5%), TTA, mouthfeel, appearance, flavor, and taste. In contrast, yoghurt produced with corn starch produced the most desirable overall acceptability, viscosity, total solids at 1.0%, and TVC (at 0.5%) concentration.

## Graphical abstract

In this paper, we reported the findings of original work on the impact of different stabilizers and fermentation time on the nutritive value of short set yoghurt. The essential textural characteristics of yoghurt are firmness and the ability to retain water, which is a factor of the type and concentration of stabilizers used. Stabilizers used in yoghurt production are many and varied. However, there is little information on how some of the stabilizers locally used in Nigeria influence the fermentation rate of yoghurt and, consequently, the nutritive value for which yoghurt is consumed. The findings from this research gave optimal concentrations of stabilizers for short set yoghurt production, which will assist manufacturers in short set yoghurt production.

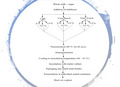

## 1 INTRODUCTION

Yoghurt is one of the most popular fermented dairy products, and it is a semisolid milk product and the best known of all fermented milk products with increasing consumption worldwide (Shiby and Mishra, 2013). It is obtained by the souring of milk using a pure culture of a particular strain of *Lactobacillus* or a mixed culture of *bulgaricus* and *Streptococcus thermophilus* and *Lactobacillus bulgaricus* in a 1:1 ratio (Sansawal et al., 2017). It can be manufactured from fresh animal liquid cow milk. In recent times, powdered cow milk is being used and vegetable milk (Soy milk) as a major base material (Obiora et al., 2020). Lactic acid and the other compounds formed during the fermentation of milk make yoghurt a food product that is acidic and creamy, appreciated for its taste and nutritional qualities, notably for its calcium content (Widayat et al., 2020). Yoghurt is thus a very convenient food as compared to very fragile milk. Due to the health benefits and taste, it constitutes an appreciable proportion of total daily food consumption or even just as a refreshing beverage in several countries (Khan et al., 2011). It is regarded as a nutritionally balanced food, containing almost all the nutrients present in milk and a more assimilable form (Olugbuyiro & Oseh, 2011). Yoghurt is a source of highly nutritive protein, energy from added cane sugar, milk fat and unfermented lactose, and vitamins (Ihekoronye & Ngoddy, 1985). It is more nutritive than milk in terms of vitamin content, digestibility, and as a source of calcium and phosphorus (Foissy, 1983). It is believed that yoghurt has valuable “therapeutic properties” and helps in curing gastrointestinal disorders (Bhattarai and Das, 2016). It also prevents and controls diarrhea, can modulate the inflammatory response produced by carcinogens, and helps in reducing the inflammatory response through an increase in apoptosis. Yoghurt is a smooth viscous smooth gel with a distinct taste of sharp acid and green apple flavor (Chen et al., 2017). Some yoghurts have a heavy consistency like custard or milk pudding, whereas others are purposely soft boiled and practically drinkable (Weerathilake et al., 2014). Firmness and smoothness are two of yoghurt's most important essential textural characteristics. The type of culture used is also an important factor in determining the microstructure and the textural properties of yoghurt (Lee & Lucey, 2010). Yoghurt is classified primarily according to its chemical composition (full‐fat, reduced‐fat, and low‐fat), manufacturing method (set and stirred yoghurt), flavor type, and postincubation process. Yoghurt is made using the method of production before incubation, cooling, and straining, and a firm jelly‐like texture characterizes it.

On the other hand, short set yoghurt is a type of yoghurt produced under controlled incubation and fermentation at 42℃ for 0 to 5 hr, improving textural properties and preventing wheying‐off defects. In comparison, long set yoghurt is a type of yoghurt produced and fermented at 30℃ for 0 to 16 hr (Abayneh, 2020).

Stabilizers and thickeners are essential in several manufactured products and dairy products such as chocolate dressing, milk drinks, ice cream, and yoghurt. These substances prevent the separation of various ingredients, increase the viscosity, and inhibit the formation of large crystals. Substances used as stabilizers and thickeners include vegetable or tree gums such a gum tragacanth and gum arabic (also known as gum acacia), agar, corn starch, gelatin, and pectin. Cellulose compounds such as methylcellulose and CMC (sodium carboxyl methyl cellulose) are also used (Bakirci and Macit, 2017). Much research has been carried out on yoghurt in terms of the final product but not so much on the effect of these stabilizers locally for yoghurt production during fermentation and on the nutritional composition of the final product. Stabilizers used in yoghurt production are many and varied. However, there is little information on how some of the stabilizers locally used in Nigeria influence the fermentation rate of yoghurt and the nutritive value for which yoghurt is consumed. From previous research, it is noteworthy to say that fermentation increases the vitamin content of products, especially some B‐complex vitamins, due to microbial activities during fermentation, where synthesis and breakdown of substances occur (Nkhata et al., 2018). Yoghurt starter cultures utilize some vitamins present in milk during fermentation for their growth. However, this increment depends on the inoculation rate, the strain of yoghurt starter cultures, and the fermentation conditions (Sharma et al., 2020). Stabilizers or hydrophilic colloids bind water, prevent separation of various ingredients, increase the viscosity, and inhibit the formation of large crystals, which are attributes for consumer acceptability.

It is, therefore, necessary to rebuild yoghurt with stabilizers and thickeners at such concentrations that will give the desired body to the final product. This goal will be achieved by optimum selection of stabilizers with protective colloid properties, by assessing how the activities of the fermenting organisms will be enhanced or inhibited by the hydrocolloids used concerning vitamin synthesis by evaluating the chemical, microbiological, nutritional, and sensory properties of yoghurt produced under controlled incubation fermented at 42℃ for 5 hr. The objectives of this work were to produce short set yoghurt with different stabilizers at different concentrations and determine the effects of these stabilizers and length of fermentation on the nutritive value of short set yoghurt.

## 2 MATERIALS AND METHODS

### 2.1 Raw materials

The raw materials used in producing the short set yoghurt samples were purchased at Ogige primary market in Nsukka Local Government Area of Enugu State, Nigeria. These include milk (peak), granulated sugar, starter culture (Yoghurmet), and stabilizers (carboxyl methylcellulose, corn starch, and gum acacia).

### 2.2 Sample preparation

#### 2.2.1 Preparation of short set Yoghurt mix

Short set yoghurt was produced according to the method of Lee and Lucey (2010) with slight modification. Dried milk sample (250 g) and granulated sugar (10 g) were weighed and made up to two liters (2 L) with clean water to produce an equivalence of fresh milk. The mixture was divided into six equal parts each for the six concentrations of the three stabilizers (CMC, corn starch, and gum acacia) added into the yoghurt mix. The stabilizers were added at a concentration of 0.5% and 1%, respectively. A control sample with a 0% stabilizer was also produced. Each yoghurt mix was pasteurized at 80℃ for 60 s, cooled to 43℃, inoculated with 2% starter culture (Yoghurmet) consisting of *Streptococcus thermophilus* and *Lactobacillus bulgaricus,* and packed into plastic containers of two liters (2 L) of capacity and allowed to ferment for 5 hr at 45℃ from where samples were withdrawn for analyses at intervals of 1 hr.

### 2.3 Production of Stabilized Yoghurt Samples

**FIGURE 1:**
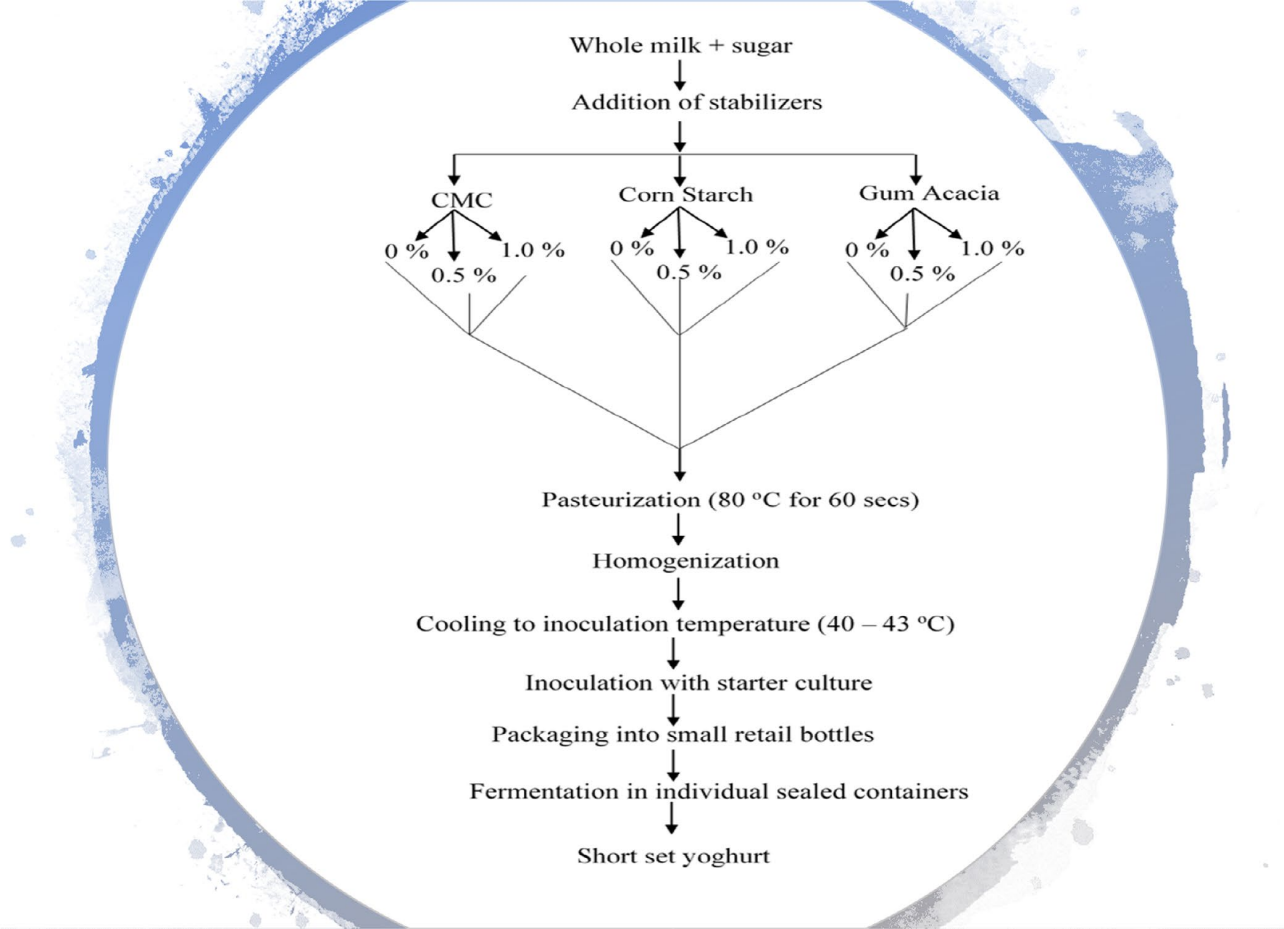
The flow chart for the production of stabilized short set yoghurt. Source: Adapted (Early, 1998)

Each yoghurt mix with its stabilizer proportion was pasteurized at 80℃ for 60 s to hasten hydration and solubilization of the solid ingredients and, more importantly, destroy organisms present in the mix (Figure 1).

This was followed by homogenization of the yoghurt sample using stainless steel, Mariam^@^ 6 in 1 blender, German Model No: M2 (1800W power), which helped to homogenize all the ingredients, especially the stabilizers and also helped to break down fat globules in milk into smaller more consistently dispersed particles, which gave a smoother and creamier product. Cooling to inoculation temperature of 40–43℃ allowed to achieve the cooling effect suitable for the culture. Inoculation with starter culture was done with 2% starter culture (*Streptococcus thermophilus* and *Lactobacillus bulgaricus*). After inoculation with a starter culture, each sample was divided into two portions of one liter each. Each one liter of the sample was dispensed into ten incubation plastic bottles of 100 ml. The first set of 1 liter from each sample was used to produce a short set yoghurt by incubating at 40–43℃ for 5 hr. Soon after homogenizing with a starter culture (Yoghurmet), samples were withdrawn from each portion of 1 liter for analyses for the zero hour of incubation. After that, samples were withdrawn at intervals of 1 hr within the fermentation period of 5 hr for short set yoghurt.

### 2.4 Proximate analyses

#### 2.4.1 Determination of moisture content

The moisture content of the samples was determined according to the standard method of the Association of Official Analytical Chemists (AOAC, 2010). The crucibles were washed thoroughly and dried in the oven (GALLEMKAMP Oven Model: REX—C900) at 100℃ for 1 hr. The hot‐dried crucibles were cooled in a desiccator (Laboware Plastic Vacuum, Desiccator, Capacity: 150 mm) and then noted down (W_1_). The sample (2 g) was weighed (CAMRY Electronic Weighing Balance) into the crucible (W_2_) and dried at 70℃ until a constant weight was obtained (W_3_). The moisture content of the samples was calculated as given in Equation (1).

(1) $$\%\text{Moisture Content}=\frac{W_{2}‐W_{3}}{W_{2}‐W_{1}}\times 100$$

where *W*_1_ = Initial weight of the empty crucible; *W*_2_ = weight of crucible + weight of the sample before drying; *W*_3_ = weight of dish +weight of the sample after drying.

#### 2.4.2 Determination of ash content

The ash content of the freshly prepared yoghurt samples was determined according to the standards of AOAC (2010). A preheated and cooled crucible was weighed (*W*_1_), and a 2 g sample was weighed into the preheated cooled crucible (*W*_2_). The sample was charred to remove hydrogen and oxygen and facilitate the ashing procedure on a Bunsen flame inside a fume cupboard. The charred sample in the crucible was transferred into a preheated muffle furnace (Carbolite AAF—1100) at 550℃ for 2 hr until a white or light gray ash was obtained (W_3_). It was cooled in a desiccator, weighed, and documented. The ash content of the samples was calculated using Equation (2)

(2) $$\%\text{Ash content}=\frac{W_{3}‐W_{1}}{W_{2}‐W_{1}}\times 100$$

where *W*_1_ = weight of the empty crucible; *W*_2_ = weight of crucible + sample before ashing; *W*_3_ = weight of crucible + sample after ashing.

#### 2.4.3 Determination of fat content

The fat content of the yoghurt samples was determined using the standard AOAC (2010) method. A Soxhlet extractor with a reflux condenser and a 500‐ml round bottom flask was fixed. The yoghurt sample (2 g) was weighed into a labeled thimble, and petroleum ether (300 ml) was filled into the round bottom flask. The extraction thimble was sealed with cotton wool. The Soxhlet apparatus, after assembling, was allowed to reflux for about 6 hr. The thimble was removed with care, and the petroleum ether drained into a container for reuse. When the flask was free of ether, it was removed and dried at 70℃ for 1 hr in an oven (GALLEMKAMP Oven Model: REX—C900). It was cooled in desiccators and then weighed. The fat content of the samples was calculated using Equation (3).

(3) $$\%\text{fat content}=\frac{\text{Weight of fat}}{\text{Weight of the sample}}\times 100$$

#### 2.4.4 Determination of crude fiber

The crude fiber was determined using the method described by AOAC (2010). About 5 ml of the sample (*w*_3_) was digested with 200 ml of 0.22 N H_2_SO_4_; it was filtered and washed severally and transferred into another conical flask. The mixture was then dissolved in 200 ml of 1.25% NaOH solution, boiled for 30 min, cold filtered, and washed with boiling water. The residue was dried in an oven (GALLEMKAMP Oven Model: REX—C900) at 105℃ for 2 hr, cooled in a desiccator, and weighed. It was incinerated at 550℃ for 2 hr in a muffle furnace (Carbolite AAF—1,100), cooled again in a desiccator, and weighed. The percentage of crude fiber was calculated, as shown in Equation 4.

(4) $$\%\text{Crude Fiber}=\frac{W_{2}‐W_{1}}{W_{3}}\times 100$$

where *W*_1_ = weight of the sample before incineration; *W*_2_ = weight of the sample after incineration; *W*_3_ = weight of the original sample.

#### 2.4.5 Determination of protein content

The protein content of the samples was determined according to the standard procedure of AOAC (2010) using the Kjeldahl method.

### 2.5 Digestion of the sample

The sample (2 g) was weighed into a Kjeldahl digestion flask followed by the addition of anhydrous barium sulfate (BaSO_4_) and hydrated copper (II) tetraoxosulfate (VI) as a catalyst. About 25 ml of concentrated tetraoxosulfate (VI) acid (H_2_SO_4_) was added with a few boiling chips. The flask with its content was heated in a fume chamber until a clear solution was obtained. The solution was cooled to room temperature, after which it was transferred into a 250‐ml volumetric flask and made up to the level with distilled water.

### 2.6 Distillation

The distillation unit was cleaned, and the apparatus was set up. A 100‐ml conical flask (receiving flask) containing 5 ml of 2% Boric acid (H_3_BO_4_) was placed under the condenser with the addition of 2 drops of methyl red indicator. A digest of 5 ml was pipetted into the apparatus through the small funnel, washed down distilled water, and followed by the addition of 5 ml of 60% sodium hydroxide (NaOH) solution. The digestion flask was heated until 100 ml of distillate (ammonium sulfate) was collected in the receiving flask.

### 2.7 Titration

The solution in the receiving flask was titrated with about 0.04 M hydrochloric acid (HCl) to get a pink color. The same procedure was carried out on the blank.

(5) $$\%\text{Nitrogen}=\frac{V_{s}‐V_{b}\times N_{\text{acid}}}{W}\times 0.0401\times 100$$

where *V_s_* = volume (ml) of acid required to titrate the sample; *V_b_* = volume (ml) of acid required to titrate blank; *N*_acid_ = Normality of acid (0.1 N); *W* = weight of the sample in gram.

#### 2.7.1 Determination of Carbohydrate

Carbohydrate (by difference) was determined using method described by AOAC (2010). It was calculated by getting the sum of the other proximate parameters and subtracting it from 100 as nitrogen‐free extract (NFE) as follows:

(6) $$\%\text{Carbohydrate}\left(\text{NFE}\right)=100‐\left(M+P+F+A\right)$$

where *M* = Moisture content; P = Protein; *F* = Fat; A = Ash.

### 2.8 Physicochemical analyses

#### 2.8.1 Determination of pH

A pocket‐sized pH meter (Hanna Instrument, Woonsocket, China, R10895) was used to determine the pH of the samples, according to AOAC (2010) method. Approximately 10% (w/v) of each of the yoghurt samples was mixed with CO_2_‐free distilled water, and the mixture was shaken vigorously. The pH meter was calibrated using a buffer solution of 4.0 and 7.0. After 10 min of calibration, the pH meter electrode was dipped into a prepared suspension of the samples for the pH measurement.

#### 2.8.2 Determination of titratable acidity

The total titratable acidity was determined using the AOAC (2010) method. The sample (5 ml) at 25℃ was measured into a flask and diluted twice its volume with distilled water. Phenolphthalein indicator (2 ml) was added to each yoghurt sample and titrated with 0.1 mol/L NaOH to the first permanent pink color. The acidity was reported as the percentage of lactic acid by weight, as shown in Equation (7).

(7) $$\text{Titratable acidity}\left(\%\right)=\frac{\text{Qty of NaOH}\left(\text{ml}\right)}{\text{Qty of Sample}\left(g\right)}\times 0.009\times 100$$

#### 2.8.3 Determination of total solids

The total solid content of the freshly prepared yoghurt with different concentrations of stabilizers was determined using AOAC (2010). The sample (5 g) was dried to a constant weight in a hot air oven (Gallenkamp) at 130℃. The total solid content was obtained as a percentage (%) of total solids.

(8) $$\text{TotalSolid}\left(\%\right)=\frac{\text{Weight of Dried Solid}}{\text{Weight of Sample}}\times 100$$

#### 2.8.4 Determination of apparent viscosity

The viscosity of short set yoghurt samples was determined as described by Ayernor and Ocloo (2007) with the use of Universal Torsion Viscometer (Gallenkamp Technico Compenstat, Gallenkamp Co. Ltd, England). 10% (w/v) of each of the yoghurt samples was used. The determination was done using an 11/16‐inch pendulum (no. 4) of standard wire gauge. The values obtained were converted to centipoises (cP) and recorded.

### 2.9 Microbial analyses

Microbiological analyses were carried out as described by Prescott et al., (2005). A serial dilution of the sample was done. The sample was placed at ambient temperature. The total viable count was performed at intervals of 1 hr within the fermentation period of 5 hr for short set yoghurt and 4‐hr interval fermentation period of 24 hr for a long set yoghurt. Total viable count (TVC) and mold count were determined by the pour plate method on nutrient agar and Sabouraud dextrose agar (SDA), respectively, as described by Prescott et al., (2005).

### 2.10 Sensory evaluation

According to Ihekoronye and Ngoddy (1985), the sensory evaluation was carried out using a 20‐man semitrained panelist. The panelists were instructed to indicate their preference for the samples. According to Iwe (2002), a nine‐point Hedonic scale, where 9 was the highest score and 1 was the lowest score for each characteristic such as color, flavor, mouthfeel, and overall acceptability, was used.

#### 2.10.1 Data analyses and experimental design

The study was designed in a split‐plot design using Design Expert^®^ version 11.1.2.0. The analysis of variance (ANOVA) was conducted on the obtained data using GEN STAT RELEASE 10.3 DE. The least significant difference (LSD) was used to compare the treatment means. Statistical significance was accepted at *p* < .05 (Steel & Torrie, 1980).

## 3 RESULTS AND DISCUSSION

### 3.1 Effects of stabilizers and fermentation time on the proximate composition of short set Yoghurt

Table 2 shows the effect of stabilizers and length of fermentation on the proximate composition of the short set yoghurt.

#### 3.1.1 Moisture content

The decrease in the moisture contents (from 88.54 ± 0.02% to 82.26 ± 0.03% ) of all short set yoghurt samples formulated with carboxyl methyl cellulose (CMC), corn starch, and gum acacia were statistically significant (*p* < .05) as compared to the control.

The decrease in moisture content from the start could be attributed to the fact that the fermentation increases the proportion of dry matter in the food. The concentration of vitamins, minerals, and protein appears to increase when measured on a dry weight basis (Olasupo & Okorie, 2019). All CMC‐stabilized short set yoghurt samples had lower moisture content at 0.5% and 1.0% concentration compared to samples stabilized with gum acacia, which had higher moisture content at similar concentrations. The lower moisture content could be attributed to the ability of CMC to increase the viscosity of the sample, which makes it exhibit functional properties of thickening, stabilization in agreement with Davidson (1980). On the other hand, gum acacia has higher water solubility (up to 50% w/v) and relatively low viscosity than other exudate gums (Dragnet, 2000). The interaction effect of the different stabilizers and their concentrations on the moisture content of short set yoghurt samples revealed significant differences (*p* < .05). This is indicative of the fact that the behavior of the stabilizers was not the same at different concentrations. The interaction effect showed that at 0% concentration, the different stabilizers behaved the same way, having similar proportions of moisture. However, a higher concentration of different stabilizers magnified the differences between the stabilizers in the moisture content of short set yoghurt. At 0.5% concentration, the yoghurt sample containing gum acacia had higher moisture content followed by corn starch, while yoghurt containing CMC had the least moisture content. At 1.0% concentration, a similar trend was observed, but the differences were high. This is attributable to the high water holding capacity of the stabilizer (CMC), which exhibited a higher water retention ability compared to the other stabilizers (Angor, 2016).

#### 3.1.2 Fat content

The stabilizers’ effect on the fat content of short set yoghurt samples indicated that significant (*p* < *.05*) differences were observed (Table 2). The fat content ranged from 2.65 ± 0.01% to 3.42 ± 0.05%. Short set yoghurt samples stabilized with corn starch recorded the highest values (3.11%–3.42%), while those containing gum acacia recorded the least fat content (2.65%–3.13%). This was attributable to the residual oil in corn starch. The effect of concentrations of the stabilizers on the fat content was significant (*p* < .05). The result obtained agreed with the findings of Tamime and Robinson (2007), who reported that fat contents ranging from 2.60% to 3.24% are for yoghurt types regarded as low fat and should contain less than 3.5% fat, while full‐fat yoghurt contains more than 3.5%. The short set yoghurt samples produced could, therefore, be categorized as low‐fat yoghurt. The fermentation time had a significant effect (*p* < .05) on the fat content of the stabilized short set yoghurt samples. An increase in the fat content was observed with an increase in fermentation time, and the values ranged from 2.65 ± 0.01% to 3.42 ± 0.05%. Yadav et al., (2007) studied the effect of milk fat content on the acid development during fermentation and rheological properties of plain yoghurt. The authors indicated increasing fat content as fermentation proceeded. The interaction effect of stabilizers and concentrations of the stabilized short set yoghurt's fat content indicated that the stabilizers' behaviors varied at different concentrations. Therefore, the differences between stabilizers were magnified by concentrations used. At 0% concentrations, the stabilizers had similar fat contents. However, at a 0.5% concentration, corn starch (C1) had a higher fat content than CMC, while gum acacia had the least fat content. Also, at 1.0% concentration, corn starch (C_2_) had a higher concentration than CMC, with gum acacia being the least, but the differences were wider at 1.0% concentration than at 0.5% concentration. This agrees with Angor, 2016, who stated that CMC used as an edible coating film reduced fat absorption and improved moisture retention in starchy products and poultry products. It could also be attributable to the fact that corn starch has more calories than CMC and gum acacia, which is more of edible fiber, thus the low‐fat content.

#### 3.1.3 Protein content

The different stabilizers had a significant effect (*p* < .05) on the protein content of the short set yoghurt samples. The protein values ranged from 3.06 ± 0.02% to 3.71 ± 0.02% (Table 2). The effect of CMC, corn starch, and gum acacia on the protein content revealed that CMC gave the highest protein content (3.25 to 3.71%) followed by gum acacia (3.28 to 3.62%), with corn starch having the lowest protein value (3.06 to 3.43%). This was in contrast with the findings of Alakali et al., (2007). Gum acacia gave high protein content close to that of CMC. This could be attributed to the fact that gum acacia has a covalent association with protein moieties rich in hydroxyproline, serine, and proline (Dragnet, 2000). Concentrations of the different stabilizers also had a significant effect (*p* < .05) on the protein content of the stabilized short set yoghurt samples.

The protein content significantly (*p* < .05) decreased with an increase in the concentration of the stabilizers. This result obtained corroborated with the study carried out by Alakali et al. (2007), which reported that a higher concentration of stabilizers reduces the nutritional quality of yoghurt samples by causing a reduction in the protein content of yoghurt due to the dilution effect. Protein contents significantly (*p* < .05) increased with an increase in fermentation time. This trend could be traced to the concentration of proteins in the yoghurt samples due to moisture loss, which caused an increase in other components. The range of values obtained was lower than that reported by Bibiana et al. (2014), who found out that the protein contents of other brands of yoghurt sold in Owerri, Imo state, were within the range of 3.76% to 5.08%. The interaction effect between the different stabilizers and their concentrations was found to be significant (*p* < .05) as well. This indicated that the effects of the stabilizers were not the same at different concentrations. It was observed that the 0% concentration of all the stabilizers had similar effects on the protein contents since the absence of the stabilizers did not create a dilution effect. At 0.5%, short set yoghurt samples containing CMC had higher protein content than those stabilized with corn starch. Also, at 1.0% concentration, a similar trend was observed. However, the differences in protein content were wider and smaller at 1% concentration due to more significant moisture loss and increased dilution effect at 1.0% concentration. Fermented milk products are good high‐quality protein sources with high biological value (Canadian Dairy Commission, 2007). Therefore, it was observed that the dilution effect caused differences in the type and quantity of stabilizers used. It was observed that gum acacia gave high protein content at 1.0% concentration due to higher reaction rates; a higher quantity of protein was observed at the higher temperature of incubation for short set yoghurt.

#### 3.1.4 Ash contents

The addition of CMC, corn starch, and gum acacia, each at levels of 0%, 0.5%, and 1.0% concentrations, caused significant differences (*p* < .05) between the ash contents of short set yoghurt samples to which different stabilizers were added. CMC recorded the highest percent ash content, presumably due to the high sodium content of CMC (Benyounes, 2012). The effect of different concentrations (0%, 0.5%, and 1.0%) of the stabilizers was found to be significant (*p* < .05) for the short set yoghurt samples. The ash content was highest at 1.0% concentration and lowest at 0% concentration due to the higher quantity of stabilizer at 1.0% concentrations. It was observed that percent ash content generally increased with an increase in fermentation time. This increase was found to be highly significant (*p* < .05) due to the concentration effect resulting from moisture loss. There was increase in ash content from 0.34 ± 0.02% to 0.79 ± 0.01% as fermentation time progressed (Table 1). The values obtained could be compared with the range given by Mbaeyi and Awaziem (2007), who reported yoghurt to contain ash from 0.49% to 0.98%. The interaction effects between the three different stabilizers and their concentrations were found to be significant (*p* < .05) (Table 2). This was in contrast with the reported work of Alakali et al., (2007). This significant interaction suggested that the differences in ash content due to stabilizers varied at different concentrations.

**TABLE 1:** Ingredient mixers for the production of short set yoghurt samples

Sample code	Stabilizer	Stabilizer concentrations g (%)	Liquid milk^a^ (ml)	Starter culture^b^ (g)	Sugar (g)
A	Control (No stabilizer)	0.00	2000	10	10
B1	CMC	10 g (0.5%)	2000	10	10
B2		20 g (1.0%)	2000	10	10
C1	Corn Starch	10 g (0.5%)	2000	10	10
C2		20 g (1.0%)	2000	10	10
D1	Gum Acacia	10 g (0.5%)	2000	10	10
D2		20 g (1.0%)	2000	10	10

^a^ Liquid milk produced by dissolving 250 g powdered milk + 10 g of sugar and made up to 2 L with water.

^b^ Starter culture = Yoghurmet.

**TABLE 2:** Effect of fermentation period (hours) on the proximate properties of short set Yoghurt

Proximate parameters	Sample	Fermentation period (hr)					
		0	1	2	3	4	5
Moisture	A	88.54 ± 0.02^a^	87.59 ± 0.33^b^	86.81 ± 0.53^b^	84.33 ± 1.13^c^	82.63 ± 0.02^d^	82.46 ± 0.03^d^
	B_1_	84.77 ± 0.02^a^	84.65 ± 0.02^b^	84.29 ± 0.02^c^	84.12 ± 0.03^d^	83.78 ± 0.02^e^	83.40 ± 0.03^f^
	B_2_	84.41 ± 0.02^a^	84.29 ± 0.02^b^	84.09 ± 0.02^c^	83.85 ± 0.02^d^	83.26 ± 0.03^e^	82.78 ± 0.03^f^
	C_1_	85.63 ± 0.02^a^	85.20 ± 0.02^b^	85.10 ± 0.03^c^	84.85 ± 0.02^d^	84.59 ± 0.02^e^	83.62 ± 0.03^f^
	C_2_	85.43 ± 0.02^a^	84.75 ± 0.02^b^	84.21 ± 0.02^c^	84.15 ± 0.03^d^	82.79 ± 0.02^e^	82.26 ± 0.03^f^
	D_1_	86.97 ± 0.02^a^	86.44 ± 0.02^b^	85.76 ± 0.03^c^	85.51 ± 0.03^d^	84.73 ± 0.03^e^	84.27 ± 0.03^f^
	D_2_	86.43 ± 0.02^a^	86.41 ± 0.03^a^	86.33 ± 0.03^b^	85.24 ± 0.03	84.59 ± 0.09^d^	84.23 ± 0.03^e^
Fat	A	3.12 ± 0.04^c^	3.13 ± 0.02^c^	3.15 ± 0.02^c^	3.25 ± 0.01^b^	3.28 ± 0.02^ab^	3.33 ± 0.05^a^
	B_1_	2.93 ± 0.01^c^	2.92 ± 0.04^c^	2.94 ± 0.03^c^	3.07 ± 0.05^b^	3.11 ± 0.02^b^	3.23 ± 0.02^a^
	B_2_	2.98 ± 0.02^de^	2.97 ± 0.02^de^	3.04 ± 0.06^d^	3.16 ± 0.04^c^	3.26 ± 0.02^b^	3.35 ± 0.05^a^
	C_1_	3.11 ± 0.02^b^	3.12 ± 0.02^b^	3.15 ± 0.02^b^	3.21 ± 0.04^a^	3.25 ± 0.02^a^	3.25 ± 0.01^a^
	C_2_	3.18 ± 0.02^d^	3.20 ± 0.01 cd	3.24 ± 0.04 cd	3.25 ± 0.05^c^	3.34 ± 0.02^b^	3.42 ± 0.05^a^
	D_1_	2.67 ± 0.02 cd	2.65 ± 0.01^e^	2.65 ± 0.02^d^	2.70 ± 0.01^bc^	2.73 ± 0.02^b^	2.85 ± 0.06^a^
	D_2_	2.70 ± 0.06^c^	2.71 ± 0.02^c^	2.70 ± 0.01^c^	2.85 ± 0.02^b^	2.92 ± 0.11^b^	3.13 ± 0.01^a^
Protein	A	3.37 ± 0.03^d^	3.36 ± 0.04^d^	3.38 ± 0.02 cd	3.43 ± 0.02^c^	3.59 ± 0.02^b^	3.67 ± 0.06^a^
	B_1_	3.35 ± 0.04^c^	3.32 ± 0.02^bc^	3.33 ± 0.03^b^	3.64 ± 0.03^a^	3.68 ± 0.01^b^	3.71 ± 0.02^a^
	B_2_	3.31 ± 0.02^c^	3.28 ± 0.04^c^	3.34 ± 0.04^c^	3.43 ± 0.02^b^	3.46 ± 0.01^a^	3.53 ± 0.05^a^
	C_1_	3.13 ± 0.03^c^	3.18 ± 0.02^c^	3.26 ± 0.05^b^	3.31 ± 0.02^b^	3.43 ± 0.04^a^	3.47 ± 0.03^a^
	C_2_	3.06 ± 0.02^d^	3.12 ± 0.04^c^	3.12 ± 0.03^c^	3.28 ± 0.02^b^	3.34 ± 0.04^a^	3.34 ± 0.03^a^
	D_1_	3.33 ± 0.04^d^	3.32 ± 0.03^d^	3.33 ± 0.04^d^	3.43 ± 0.02^b^	3.56 ± 0.03^b^	3.62 ± 0.04^a^
	D_2_	3.29 ± 0.02	3.28 ± 0.05^c^	3.29 ± 0.02^c^	3.34 ± 0.05^bc^	3.39 ± 0.03^ab^	3.44 ± 0 0.03^a^
Ash	A	0.34 ± 0.02^e^	0.54 ± 0.02^d^	0.56 ± 0.02^d^	0.61 ± 0.01^c^	0.65 ± 0.02^b^	0.69 ± 0.02^a^
	B_1_	0.65 ± 0.05^bc^	0.58 ± 0.03^c^	0.63 ± 0.04^bc^	0.70 ± 0.01^ab^	0.75 ± 0.05^a^	0.76 ± 0.03^a^
	B_2_	0.61 ± 0.02^b^	0.62 ± 0.03^b^	0.65 ± 0.04^b^	0.76 ± 0.04^a^	0.78 ± 0.02^a^	0.79 ± 0.01^a^
	C_1_	0.56 ± 0.04^b^	0.57 ± 0.05^b^	0.57 ± 0.02^a^	0.66 ± 0.03^a^	0.69 ± 0.01^a^	0.71 ± 0.04^a^
	C_2_	0.56 ± 0.03^a^	0.59 ± 0.08^a^	0.59 ± 0.02^a^	0.68 ± 0.01^a^	0.66 ± 0.01^a^	0.69 ± 0.02^a^
	D_1_	0.47 ± 0.05^c^	0.53 ± 0.03^bc^	0.54 ± 0.03^b^	0.63 ± 0.01^a^	0.68 ± 0.01^a^	0.66 ± 0.04^a^
	D_2_	0.50 ± 0.02^b^	0.52 ± 0.04^b^	0.52 ± 0.04^b^	0.64 ± 0.05^a^	0.69 ± 0.01^a^	0.69 ± 0.00^a^

Note

Mean ± *SD* of triplicate readings.

Values with a different superscript in the same column are significantly different.

Keys: Fermentation period: 0–5 hr; Sample A = Short set yoghurt without any stabilizer; Sample B_1_ = Short set yoghurt with 0.5% CMC; Sample B_2_ = Short set yoghurt with 1.0% CMC; Sample C_1_ = Short set yoghurt with 0.5% Corn starch; Sample C_2_ = Short set yoghurt with 1.0% Corn starch; Sample D_1_ = Short set yoghurt with 0.5% Gum acacia; Sample D_2_ = Short set yoghurt with 1.0% Gum acacia.

### 3.2 Effects of stabilizers and fermentation time on the physicochemical properties of short set Yoghurt

Tables 3 and 4 show the effect of stabilizers (type and concentration) and length of fermentation on the physicochemical properties of the short set yoghurt samples.

**TABLE 3:** Physicochemical properties of Short Set Yoghurt Samples at different fermentation period

Physicochemical parameters	Sample	0	1	2	3	4	5
pH	A	6.19 ± 0.03^a^	6.18 ± 0.03^a^	6.18 ± 0.01^a^	5.09 ± 0.03^b^	4.52 ± 0.02^c^	4.18 ± 0.02^d^
	B_1_	6.27 ± 0.01^a^	6.26 ± 0.01^a^	6.25 ± 0.02^a^	5.16 ± 0.04^b^	4.85 ± 0.04^c^	4.15 ± 0.04^d^
	B_2_	6.25 ± 0.02^a^	6.25 ± 0.02^a^	6.23 ± 0.02^a^	5.23 ± 0.01^b^	4.97 ± 0.03^c^	4.20 ± 0.01^d^
	C_1_	6.18 ± 0.01^a^	6.17 ± 0.01^a^	6.17 ± 0.03^a^	5.06 ± 0.05^b^	4.84 ± 0.04^c^	4.45 ± 0.03^d^
	C_2_	6.17 ± 0.03^a^	6.16 ± 0.02^a^	6.13 ± 0.03^a^	5.09 ± 0.02^b^	4.93 ± 0.03^c^	4.45 ± 0.03^d^
	D_1_	5.99 ± 0.04^a^	6.96 ± 0.02^a^	5.84 ± 0.02^b^	5.76 ± 0.04^c^	4.96 ± 0.02^d^	4.10 ± 0.03^e^
	D_2_	5.89 ± 0.02^d^	5.87 ± 0.05^ab^	5.84 ± 0.02^bc^	5.81 ± 0.02^c^	4.49 ± 0.02^d^	4.14 ± 0.04^e^
TTA	A	0.29 ± 0.01^d^	0.30 ± 0.01 cd	0.31 ± 0.00^d^	0.63 ± 0.00^b^	0.75 ± 0.00^a^	0.75 ± 0.00^a^
	B_1_	0.29 ± 0.01^e^	0.30 ± 0.00^de^	0.31 ± 0.00^d^	0.52 ± 0.00^c^	0.55 ± 0.02^b^	0.69 ± 0.00^a^
	B_2_	0.48 ± 0.00^e^	0.48 ± 0.00^e^	0.54 ± 0.00^d^	0.56 ± 0.00^c^	0.59 ± 0.00^b^	0.69 ± 0.00^a^
	C_1_	0.36 ± 0.00^d^	0.39 ± 0.00^c^	0.39 ± 0.00^c^	0.59 ± 0.00^b^	0.60 ± 0.01^b^	0.70 ± 0.04^a^
	C_2_	0.28 ± 0.01^f^	0.30 ± 0.01^e^	0.39 ± 0.00^d^	0.52 ± 0.00^c^	0.59 ± 0.00_b_	0.70 ± 0.01^a^
	D_1_	0.55 ± 0.00^e^	0.57 ± 0.03^e^	0.61 ± 0.00^d^	0.65 ± 0.00^c^	0.75 ± 0.00^b^	0.84 ± 0.00^a^
	D_2_	0.34 ± 0.00^e^	0.35 ± 0.00^e^	0.36 ± 0.00^d^	0.69 ± 0.00^c^	0.79 ± 0.00^b^	0.89 ± 0.02^a^
Viscosity	A	1.48 ± 0.03^e^	8.17 ± 0.05^d^	8.87 ± 0.32^d^	103.92 ± 1.52^c^	197.89 ± 1.06^b^	119.62 ± 0.70^a^
	B_1_	58.31 ± 1.79^e^	96.66 ± 1.36^e^	98.74 ± 1.43^d^	154.30 ± 3.21^c^	166.27 ± 2.97^b^	264.00 ± 2.39^a^
	B_2_	97.74 ± 0.49^e^	105.06 ± 0.84^d^	106.41 ± 0.90^d^	161.76 ± 1.04^c^	169.88 ± 1.58^b^	275.57 ± 4.08^a^
	C_1_	2.45 ± 0.04^e^	784.00 ± 0.01^d^	8.45 ± 0.05^d^	120.62 ± 0.48^c^	128.45 ± 0.78^b^	239.51 ± 1.81^a^
	C_2_	3.02 ± 0.04^d^	8.10 ± 0.03^c^	8.55 ± 0.03^c^	130.60 ± 0.77^b^	130.65 ± 0.82^b^	248.47 ± 1.26^a^
	D_1_	1.49 ± 0.03^e^	4.85 ± 0.25^d^	5.99 ± 0.13^d^	90.82 ± 1.42^c^	106.26 ± 1.54^b^	120.68±429^a^
	D_2_	2.5 ± 0.03^e^	5.19 ± 0.02^d^	6.18 ± 0.03^d^	101.36 ± 1.28^c^	111.00 ± 1.20^b^	126.49 ± 2.93^a^
Total Solid	A	11.64 ± 0.02^d^	12.41 ± 0.35^c^	13.91 ± 0.53^c^	15.67 ± 1.13^b^	17.37 ± 0.02^a^	17.54 ± 0.03^a^
	B_1_	15.23 ± 0.02^f^	15.35 ± 0.02^e^	15.70 ± 0.02^d^	15.88 ± 0.03^c^	16.22 ± 0.02^a^	16.60 ± 0.03^a^
	B_2_	15.59 ± 0.02^f^	15.71 ± 0.02^e^	15.90 ± 0.02^d^	16.36 ± 0.03^c^	16. 74 ± 0.03^b^	17.22 ± 0.03^a^
	C_1_	14.37 ± 0.02^d^	14.80 ± 0.02^c^	14.89 ± 0.03^c^	15.15 ± 0.02^bc^	15.41 ± 0.02^b^	16.05 ± 0.58^a^
	C_2_	14.57 ± 0.02^f^	15.25 ± 0.02^e^	15.79 ± 0.02^d^	15.85 ± 0.03^c^	17.44 ± 0.04^b^	17.74 ± 0.03^a^
	D_1_	13.03 ± 0.02^f^	13.56 ± 0.02^e^	14.24 ± 0.03^d^	14.49 ± 0.03^c^	15.27 ± 0.03^b^	15.74 ± 0.03^a^
	D_2_	13.57 ± 0.02^d^	13.59 ± 0.03^d^	13.67 ± 0.03^d^	14.76 ± 0.03^c^	15.40 ± 0.09^b^	15.70 ± 0.13^a^

Notes

Mean ± *SD* of triplicate readings. Values with different superscripts in the same column are significantly different. Keys: Fermentation period: 0–5 hr; Sample A = Short set yoghurt without any stabilizer; Sample B_1_ = Short set yoghurt with 0.5% CMC; Sample B_2_ = Short set yoghurt with 1.0% CMC; Sample C_1_ = Short set yoghurt with 0.5% Corn starch; Sample C_2_ = Short set yoghurt with 1.0% Corn starch; Sample D_1_ = Short set yoghurt with 0.5% Gum acacia; Sample D_2_ = Short set yoghurt with 1.0% Gum acacia.

**TABLE 4:** Rate of fermentation of short set yoghurt samples

Short Set yoghurt (SSY)_Stabilizers	Conc. of stabilizer (%)	MC (%/hr)	Viscosity (cP/hr)	TTA (%/hr)	pH (unit/hr)	TVC (cfu/ml/hr)	LAB (cfu/ml/hr)	Vit. B_3_ mg/ml/hr
SSY_CMC	0	1.36	28.13	0.12	0.46	0.65	0.36	0.08
	0.5	0.28	36.94	0.09	0.45	1.76	0.6	0.02
	1.0	0.33	32.54	0.04	0.43	0.13	0.34	0.01
SSY_Corn Starch	0	1.36	28.13	0.12	0.46	0.65	0.36	0.08
	0.5	0.35	47.41	0.07	0.39	0.58	0.32	0.01
	1.0	0.62	20.48	0.09	0.37	0.53	0.44	0.01
SSY_Gum Acacia	0	1.36	28.13	0.12	0.46	0.65	0.36	0.08
	0.5	0.54	28.14	0.06	0.36	0.77	1.28	0.02
	1.0	0.50	29.52	0.13	0.37	0.90	0.27	0.02

Note

Mean ± *SD* of triplicate readings. Values with a different superscript in the same column are significantly different. Keys: MC, Moisture content; TVC, Total viable count; LAB, Lactic acid bacteria; TTA, Total titratable acidity; Vit. B_3_, Niacin content.

#### 3.2.1 pH

The short set yoghurt samples containing 0%, 0.5%, and 1.0% of CMC, corn starch, and gum acacia indicated that the differences between effects of stabilizers were not significant between CMC and corn starch but differed significantly (*p* < .05) from gum acacia. Although titratable acidity production in yoghurt containing corn starch was higher than yoghurt containing CMC (Table 4), the pH of the yoghurt samples containing CMC and corn starch was similar, suggesting that the pH of yoghurt containing corn starch had a higher buffering capacity. This was unlike the yoghurt containing gum acacia, which appeared to be less buffered. However, CMC gave the highest pH value when compared to the other stabilizers. This could be attributed to the fact that CMC is a stabilizer that is more soluble in alkali conditions and insoluble in acidic conditions and has an optimum pH range of 6.0–8.5 (1 in 100 solutions) (Dragnet, 2000). It could also be attributable to a lower level of fermentation of CMC. It gave a higher pH value than corn starch and gum acacia, which was somehow more fermented. The differences caused by the concentration of the stabilizers on the pH value of short set yoghurts were statistically significant (*p* < .05). This means that the pH generally decreased with an increase in the concentration of the stabilizers, but the nature of the decrease was significantly (*p* < .05) different for different stabilizers. The pH of the yoghurt with no stabilizer 0% (control) differed significantly from those of 0.5% and 1.0% concentrations. The highest pH was produced at 0.5% concentration by CMC and corn starch. The lowest pH was produced at 0.5% concentration by gum acacia. This low pH value of gum acacia could be as a result that gum acacia as a stabilizer has an optimum pH range of 4.5 (William and Phillips, 2009), which lowered the pH of the yoghurt prepared with this stabilizer. It could also be attributed to the stability of gum acacia in acid conditions and high solubility (Eqbal & Abdullah, 2013) or that gum acacia was also fermented along with lactose and possesses lower buffering capacity. The decrease in pH as the concentration rises may be attributable to the continued fermentation of the lactic acid bacteria and the acidity effect of the added stabilizers (Ibrahim & Khalifa, 2015). Fermentation time had a significant (*p* < .05) effect on the pH value obtained for short set yoghurt samples. The pH values decreased (6.27 ± 0.01 to 4.10 ± 0.03) as fermentation time increased. This decrease in pH as fermentation progressed could be attributed to the increased and sustained metabolic activity of acid‐producing microorganisms (Gassem & Abu‐Tarboush, 2000), resulting in the continued production of lactic acid with consequent depression of pH. The result also revealed a significant (*p* < .05) difference in the interaction effect between the different stabilizers and their concentrations. The interaction effect between stabilizers and concentration for the short set yoghurt samples at 0.5% and 1.0% concentrations did not magnify much difference between the stabilizers. Due to higher reaction rates of yoghurt produced with the short set method, the final pH produced in 5 hr was below 4.5.

#### 3.2.2 Total titratable acidity (TTA)

Titratable acidity values of the short set yoghurt samples containing CMC, corn starch, and gum acacia at 0%, 0.5%, and 1.0% concentrations showed significant differences (*p* < .05) between the stabilizers, with gum acacia giving the highest values for TTA (Table 3). The differences caused by concentrations of stabilizers on the short set were statistically significant (*p* < .05). It was observed that the addition of CMC at the level of 1.0% concentration depressed the production of lactic acid. This was clearly shown in Table 4 by the production rate of lactic acid in the presence of a high level of CMC. It was shown that at 0% concentration, the production rate of lactic acid was 0.12% per hour, but this depressed to 0.09% per hour when CMC increased to 0.5%. When CMC increased to 1% concentration, the lactic acid production depressed further to 0.04% per hour.

CMC is an anionic, water‐soluble polymer capable of forming a very viscous solution. CMC is insoluble in acidic conditions and more soluble in alkali conditions, and the solubility is pH‐dependent (Ergun et al., 2016). Therefore, the low acid production could be attributed to its formation of highly viscous systems, which caused diffusion resistance that reduced mobility of reactants and the consequence was the reduction of the rate at which the reacting species came together for fermentation to take place (Alakali et al., 2007). It could also be observed from Table 4 that significant interaction between stabilizers and concentrations of the different stabilizers suggests that the different concentrations of the stabilizers magnified the differences in the niacin content between the stabilizers. At 1.0% concentration, the rate of elaboration of niacin reduced further and maintained the differences observed at 0.5% concentration. Gum acacia did not impede the production of titratable acidity. Instead, the higher concentration of gum acacia resulted in a higher lactic acid production rate (Table 4). It was seen that at 0.5% concentrations, the rate of lactic acid production by gum acacia was 0.01% per hour, while at 1.0% concentration, it increased to 0.05% per hour. Gum acacia is a stabilizer that functions as an emulsifying agent in milk products by producing a firmer texture (Roeper, 2014). Gum acacia has high water solubility (up to 50%. w/v) and relatively low viscosity than other exudate gums. This polymer's highly branched molecular structure and relatively low molecular weight are responsible for these properties (Dragnet, 2000). The low viscosity of gum acacia allowed greater freedom of mobility of reactants, which enabled reacting species to come together for fermentation to take place. The titratable acidity of the short set yoghurt samples stabilized with corn starch at the level of 0%, 0.5%, and 1.0% concentrations had a less inhibitory effect on the production of titratable acidity compared to CMC at similar concentrations. It was seen from Table 4 that the rate of change at 0.5% concentration was 0.07% and 0.09% per hour in the short set. Therefore, the result shows that corn starch in concentrations beyond 0.5% and 1.0% could be wastage unless used in combination with other stabilizers with lower viscous properties. It is possible that whereas the lactic acid bacteria were not fermenting CMC, they could ferment corn starch and gum acacia to some extent.

Hence, the higher concentration of corn starch and gum acacia resulted in a higher rate change. The fermentation time had a significant effect (*p* < .05) on the total titratable acid of short set yoghurt samples. It was observed that TTA improved as fermentation time progressed. The TTA values for short set yoghurt ranged from 0.29 ± 0.01% to 0.89 ± 0.02%. This result agreed with those previously reported for other Labnehs (a strained yoghurt used for sandwiches in an Arab country) (Benkerroum and Tamime, 2004). It also agreed with the findings of Ahmad (1994), who reported that the total titratable acidity ranged from 0.87% to 1.13%. The interaction effect of stabilizers and concentrations was significant (*p* < .05), suggesting that the effects caused by different stabilizers used were different at different concentrations.

#### 3.2.3 Viscosity

The viscosity values obtained for the short set yoghurt samples stabilized with CMC, corn starch, and gum acacia at 0, 0.5, and 1.0% concentrations showed that significant differences (*p* < .05) were found between the different stabilizers (Table 3). The differences in viscosities have been attributed to the chemical and physical characteristics of the stabilizers used. CMC can form high viscous colloidal solutions with water, insoluble in ethanol and slightly hygroscopic (Dragnet, 2000). Corn starch can disperse and suspend other ingredients or particulate matter, thereby forming gels and provides the body with food products (Erickson, 2006). On the other hand, gum acacia dissolves easily in water (up to 50%), and the resulting solution does not interact easily with other chemical compounds (ITC, 2008). Therefore, a comparison of the viscosity of gum acacia with sodium carboxyl methylcellulose, a common thickening agent, showed that even at a concentration above 30%, gum acacia solution has a lower viscosity than 1.0% sodium carboxyl methylcellulose at low shear rates. Also, while gum acacia is Newtonian in behavior with viscosity being shear rate‐independent, sodium carboxyl methylcellulose displayed non‐Newtonian shear thinning characteristics (Williams & Phillips, 2009).

The viscosity of gum acacia decreased in the presence of electrolytes due to charge screening and at low pH when the carboxyl groups become undissociated (Williams & Phillips, 2009). At 1.0% concentration, CMC recorded the highest viscosity compared to other stabilizers. Viscosity increased significantly (*p* < .05) with an increase in the concentration of each stabilizer, with CMC having the greatest effect. CMC increased from 58.33 ± 1.79 cP at 0% concentration to 152.74 ± 3.21 cP at 1.0% concentration, corn starch increased from 58.33 ± 1.79 cP to 88.23 ± 0.05 cP, and gum acacia increased from 58.33 ± 1.79 cP to 58.76 ± 0.08 cP. The rate of increase in viscosity of short set yoghurt, as shown in Table 3, revealed that the increase in viscosity caused by CMC and corn starch peaked at 0.5% concentration. Therefore, further addition of CMC and corn starch would not increase the viscosity of short set yoghurt further. Gum acacia showed a linear relationship, and this implies that as the concentration of the stabilizer increases, viscosity increases. It was observed that there was an increase in viscosity of short set yoghurt as the fermentation time increased.

#### 3.2.4 Total solids

The values presented in Table 4 showed information on the total solids of short set yoghurt samples stabilized with 0%, 0.5%, and 1.0% concentrations of CMC, corn starch, and gum acacia. There was a significant (*p* < .05) difference in the total solids. Total solids were higher in the samples with stabilizers than the control yoghurt sample as the concentration increased. This trend was consistent with the report of Mehanna et al., (2013), in which the total solids, protein, and fat contents were found to be higher in stabilized yoghurt samples. The increment in the total solids was said to have resulted from the stabilizers incorporated in the samples. Samples stabilized with corn starch gave the highest total solids content (17.74 ± 0.03%) followed by CMC (17.22 ± 0.03%), while gum acacia had the least total solids contents (15.74 ± 0.03%). This could be because each polymer chain in a dilute solution of corn starch is hydrated and extended, therefore exhibiting stable consistencies (Edali et al., 2001). The total solid content increased with an increase in fermentation time. This result was in agreement with the findings of Sahid et al., (2002), who reported the total solids content with a range of 13.80% to 18.30%. This result showed that total solids accumulate as fermentation progresses because moisture was lost. The interaction effect between stabilizer and concentrations on the total solids contents was significant (*p* < .05). The increase could be attributed to the accumulation of solid matter during fermentation. CMC incorporated in the short set yoghurt samples significantly had the highest total solids compared to other stabilizers, while 1.0% concentration recorded the highest total solids compared to other concentrations. Therefore, the differences in the effect of the stabilizers were magnified by the concentrations used.

This could be attributed to the coagulation of the protein and carbohydrate during fermentation (Amankwah et al., 2009) as pH decreased. However, due to the higher reaction rate caused by the elevated incubation temperature in the short set yoghurt, higher viscosity was achieved in the short set yoghurt within 5 hr. Interaction effects between stabilizers and their concentration were found significant (*p* < .05). The behaviors of the stabilizers at different concentrations were different for different stabilizers. Thus, an increase in the concentrations of stabilizers magnified the differences in effect between stabilizers.

### 3.3 Effects of stabilizers and fermentation time on the microbial qualities of short set Yoghurt

#### 3.3.1 Total viable count

The total viable count of the short set yoghurt stabilized with CMC, corn starch, and gum acacia showed significant (*p* < .05) differences among the stabilizers. CMC and gum acacia gave the highest values of TVC and lactic acid bacteria counts compared to corn starch for short set yoghurt. Total viable count decreased significantly (*p* < .05) with increased concentration. TVC values for the short set yoghurt samples ranged from 1.30^4^ ± 2.00 to 8.30 ± 3.00 × 10^4^ (Table 5). The rate of multiplication of microorganisms indicated that at 0.5% concentration of CMC, the rate of TVC was lower when compared to 1.0% concentration of the same stabilizer. This suggests that this concentration of stabilizer (1.0%) provided the optimum conditions for the growth of the microorganisms. For corn starch and gum acacia, the rate of total viable count production decreased with an increase in the concentration of the stabilizers, apparently because they did not provide optimum conditions for the growth of the microorganisms.

**TABLE 5:** Microbial properties of short set yoghurt samples

Microbial parameters	Sample	Fermentation period (hr)					
		0	1	2	3	4	5
TVC	A	2.00 ± 2.65^d^	7.30 ± 2.00^c^	1.04 ± 2.65^b^	1.17 ± 2.56^a^	1.20 ± 2.56^a^	1.05 ± 2.65^b^
	B_1_	1.30 ± 2.00^f^	2.30 ± 2.00^e^	3.50 ± 2.65^d^	4.10 ± 2.65^c^	9.30 ± 3.46^b^	1.02 ± 3.00^a^
	B_2_	1.60 ± 3.00^d^	3.00 ± 3.00^b^	2.40 ± 3.00^c^	2.30 ± 3.00^c^	5.30 ± 3.00^a^	5.20 ± 3.00^a^
	C_1_	1.80 ± 3.00^b^	1.90 ± 3.00^b^	2.00 ± 3.00^b^	2.20 x ± 3.00^b^	4.60 ± 2.00^a^	4.20 ± 2.00^a^
	C_2_	1.30 ± 3.46^c^	2.10 ± 3.00^b^	2.20 ± 3.00^b^	2.40 ± 3.00^c^	3.90 ± 3.00^a^	4.00 ± 3.00^a^
	D_1_	2.10 ± 1.73^e^	3.50 ± 3.00^d^	4.70 ± 3.46^c^	7.90 ± 3.46^b^	1.07 ± 2.12^a^	8.30 ± 3.00^a^
	D_2_	1.80 ± 1.73^e^	2.36 ± 3.05^d^	4.50 ± 3.46^c^	4.90 ± 3.00^c^	5.50 ± 3.46^a^	5.60 ± 3.00^b^
LAB	A	2.60 ± 2.65^f^	7.60 ± 2.65^d^	1.21 ± 2.65^a^	1.12 ± 3.00^b^	9.60 ± 2.56^c^	3.70 ± 4.36^e^
	B_1_	2.0 ± 3.00^d^	3.40 x ± 3.00_c_	4.00 ± 3.00^b^	4.30 ± 3.00^b^	5.30 ± 2.00^a^	5.00 ± 3.00^a^
	B_2_	2.30 ± 3.00^d^	3.40 x ± 3.00^c^	3.57 ± 3.51^bc^	3.80 ± 2.00^bc^	4.50 ± 3.00^a^	4.00 ± 2.00^ab^
	C_1_	1.80 ± 3.00^b^	2.00 ± 3.00^b^	2.00 ± 3.00^b^	2.20 ± 3.00^b^	3.40 ± 2.00^a^	3.20 ± 1.00^a^
	C_2_	1.60 ± 3.00^d^	1.90x±3.00cd	2.30 ± 3.00^b^	2.30 ± 3.00^b^	3.70 ± 3.00^a^	3.60 ± 3.00^a^
	D_1_	2.10 ± 3.00^e^	2.50 ± 3.00^e^	3.50 ± 3.46^d^	5.50 ± 3.46^c^	6.30 ± 3.00^b^	8.40 ± 4.24^a^
	D_2_	1.80 ± 3.00^b^	2.30 ± 3.00^b^	2.90 ± 3.46^a^	3.00 ± 3.46^a^	3.30 ± 3.00^a^	3.10 ± 3.00^a^

Note

Mean ± *SD* of triplicate readings (×10^4^ CFU/ml).

Values with different superscripts in the same column are significantly different.

Keys: Fermentation period: 0‐5h; Sample A = Short set yoghurt without any stabilizer; Sample B_1_ = Short set yoghurt with 0.5% CMC; Sample B_2_ = Short set yoghurt with 1.0% CMC; Sample C_1_ = Short set yoghurt with 0.5% Corn starch; Sample C_2_ = Short set yoghurt with 1.0% Corn starch; Sample D_1_ = Short set yoghurt with 0.5% Gum acacia; Sample D_2_ = Short set yoghurt with 1.0% Gum acacia.

#### 3.3.2 Lactic acid bacteria count

LAB values for short set yoghurt samples at 0% concentration were 7.8 × 105 cfu/ml, 3.72 × 10^5^ CFU/ml at 0.5% concentration, and 2.96 × 10^5^ CFU/ml at 1.0% concentrations. The LAB count values obtained ranged from 1.12 × 10^4^ ± 3.00 to 8.40 × 10^4^ ± 4.24 CFU / ml. However, the decrease in the activity of the lactic acid bacteria caused an increase in the pH of the yoghurt samples. The rate of LAB multiplication for the short set yoghurt (Table 5) indicated that at 0.5% concentration of CMC, multiplication of LAB was faster when compared to 1.0% concentration. The rate of multiplication of LAB for corn starch and gum acacia showed a decrease with an increase in the concentration of the stabilizer. This could be because the optimum conditions for LAB proliferation were provided at a 0.5% concentration of CMC compared to other concentrations. From Table 5, it was observed that LAB proliferation for the different stabilizers at 0.5% concentration was slow compared to the rate of proliferation of LAB at 1.0% concentration; this could be attributed to the conditions of fermentation, which did not favor the rapid LAB growth. Fermentation time led to a significant (*p* < .05) increase in the total viable count of short set yoghurt. This was consistent with other reported works by Gassem and Abu‐Tarboush (2000), which reported LAB evolution in yoghurt, showing an increase in number versus fermentation time. The significant interaction effect between stabilizers and the concentrations of the different stabilizers suggested that the different stabilizers' behavior was different at different concentrations. This implies that higher concentrations magnified the differences between stabilizers in the total viable count and lactic acid bacteria count in the yoghurt samples.

### 3.4 Effects of stabilizers and fermentation time on the sensory attributes of short set Yoghurt

The sensory scores of short set yoghurt samples stabilized with CMC, corn starch, and gum acacia at 0%, 0.5%, and 1.0% concentrations are presented in Table 6. There were significant (*p* < .05) differences in the sensory parameters of the yoghurt samples from the result.

**TABLE 6:** Sensory attributes of Short Set Yoghurt Samples

Sensory parameters	Sample	Fermentation period (hr)		
		3	4	5
Color	A	6.75 ± 0.44^ab^	7.00 ± 0.00^a^	7.35 ± 0.59^a^
	B_1_	6.45 ± 0.51^c^	6.80 ± 0.41^a^	7.30 ± 0.47^a^
	B_2_	6.20 ± 0.41^b^	6.60 ± 0.59^b^	6.55 ± 0.61^a^
	C_1_	5.60 ± 0.59^c^	6.00 ± 0.34^b^	6.65 ± 0.49^a^
	C_2_	6.10 ± 0.31^c^	6.75 ± 0.44^b^	7.30 ± 0.47^a^
	D_1_	6.10 ± 0.31^b^	6.35 ± 0.49^ab^	6.60 ± 0.50^a^
	D_2_	6.25 ± 0.44^c^	6.60 ± 0.68^b^	7.45 ± 0.51^a^
Flavor	A	6.00 ± 0.00^c^	6.40 ± 0.50^b^	6.80 ± 0.41^a^
	B_1_	6.20 ± 0.41^c^	6.45 ± 0.51^b^	6.80 ± 0.41^a^
	B_2_	5.60 ± 0.50^a^	5.65 ± 0.49^a^	5.25 ± 0.72^b^
	C_1_	5.25 ± 0.55^b^	5.80 ± 0.62^ab^	6.20 ± 0.41^a^
	C_2_	5.85 ± 0.49^c^	6.55 ± 0.69^b^	7.10 ± 0.31^a^
	D_1_	5.30 ± 0.92^b^	6.20 ± 0.69^a^	6.40 ± 0.50^a^
	D_2_	6.15 ± 0.37^c^	7.00 ± 0.46^ab^	7.15 ± 0.49^a^
Taste	A	6.15 ± 0.37^c^	6.30 ± 0.47^b^	6.95 ± 0.22^a^
	B_1_	5.80 ± 0.41^c^	6.20 ± 0.41^b^	6.70 ± 0.47^a^
	B_2_	5.35 ± 0.49^a^	5.05 ± 0.61^b^	5.35 ± 0.81^a^
	C_1_	5.15 ± 0.49^a^	6.10 ± 0.64^b^	6.85 ± 0.59^a^
	C_2_	5.80 ± 0.41^c^	6.55 ± 0.51^b^	7.05 ± 0.51^a^
	D_1_	4.60 ± 1.47^c^	6.00 ± 0.97^b^	6.45 ± 0.61^a^
	D_2_	5.95 ± 0.39^b^	6.90 ± 0.72^a^	7.10 ± 0.55^a^
Mouthfeel	A	6.25 ± 0.44^b^	6.55 ± 0.51^b^	6.90 ± 0.31^a^
	B_1_	6.30 ± 0.57^c^	6.55 ± 0.51^b^	6.85 ± 0.37^a^
	B_2_	5.40 ± 0.50^b^	5.40 ± 0.68^b^	5.65 ± 0.49^a^
	C_1_	5.35 ± 0.59^b^	6.00 ± 0.32^a^	6.30 ± 0.47^a^
	C_2_	6.00 ± 0.46^b^	6.80 ± 0.41^ab^	7.00 ± 0.46^a^
	D_1_	4.80 ± 1.39^c^	6.10 ± 0.85^b^	6.20 ± 0.69^a^
	D_2_	6.10 ± 0.64^b^	7.00 ± 0.00^a^	7.40 ± 0.50^a^
Overall Acceptability	A	6.05 ± 0.22^c^	6.80 ± 0.52^b^	7.35 ± 0.49^a^
	B_1_	6.00 ± 0.31^b^	6.60 ± 0.50^b^	7.05 ± 0.39^a^
	B_2_	5.90 ± 0.31^b^	5.55 ± 0.83^c^	6.05 ± 0.69^a^
	C_1_	5.20 ± 0.41^c^	6.35 ± 0.61^b^	6.95 ± 0.22^a^
	C_2_	6.10 ± 0.55^c^	7.30 ± 0.47^b^	7.90 ± 0.31^a^
	D_1_	5.40 ± 1.09^c^	6.75 ± 0.44^b^	6.90 ± 0.31^a^
	D_2_	6.50 ± 0.51^b^	7.25 ± 0.44^a^	7.65 ± 0.49^a^

Note

Mean ± *SD* of triplicate readings.

Values with different superscripts in the same column are significantly different.

#### 3.4.1 Flavor

Table 6 showed the sensory scores of the short set yoghurt samples stabilized with CMC, corn starch, and gum acacia.

The mean score for flavor ranged from neither liked nor disliked (5.25 ± 0.55) to liked moderately (7.15 ± 0.49). Samples containing 1.0% gum acacia had the highest score for flavor. Pecivova et al., (2013) reported that the addition of gum acacia could enhance the quality and flavor of pizza flans. Notably, there was significant (*p* < .05) improvement in flavor for samples stabilized with corn starch followed by those containing gum acacia as concentration increased. However, this trend was reversed in samples containing CMC with an increase in concentration. The flavor of short set yoghurt produced with corn starch was most preferred. This could be due to starch hydrolysis, which leads to the release of sweetening properties of the starch (Erickson, 2006). There was marked significant (*p* < .05) improvement in the sensory score in each of the stabilized yoghurt samples at 1.0% concentrations. For short set yoghurt, all the sensory parameters (especially flavor) improved with an increase in the concentration of the stabilizers and as fermentation time progressed (3–5 hr). Therefore, the differences between the stabilizers could be attributed to the acidity level developed during fermentation (Alakali et al., 2007).

#### 3.4.2 Mouthfeel

The control yoghurt had higher mouthfeel scores when the fermentation was left for 3 hr compared to other treated short set yoghurt samples. As the fermentation progressed to 4 hr, gum acacia containing yoghurt samples were liked moderately (7.00 ± 0.00) at 1.0% concentration. By the time the fermentation had reached 5 hr, only those samples stabilized with 1.0% gum acacia were found to have higher mouthfeel scores than the control yoghurt; nevertheless, the improvement observed was not significant (*p* > .05).

All samples stabilized with CMC were found to score lower scores for mouthfeel than the control yoghurt samples as concentrations increased. With corn starch added to the samples, the mouthfeel scores significantly improved as the concentrations changed from 0.5% to 1.0%. A notable improvement was achieved in the mouthfeel of corn starch containing samples as the fermentation time progressed from 4 to 5 hr, compared to the samples without any stabilizer. A similar trend in corn starch was observed when gum acacia was added to the short set yoghurt samples.

#### 3.4.3 Color

The increased concentration of CMC was observed to have adversely affected the appearance (color) of short set yoghurt samples significantly (*p* < .05) in comparison with the short set yoghurt without a stabilizer. As the fermentation time increased (4 to 5 hr), the decrease in the sensory color scores so observed was no longer significant (*p* > .05).

Although samples in which corn starch was added were found to have higher sensory scores for color as the fermentation time increased (3–5 hr), the yoghurt samples without a stabilizer (control) still had higher sensory scores compared to the treated yoghurt samples.

Only samples formulated with gum acacia at the 1.0% level were scored higher in appearance than any other yoghurt samples (treated with stabilizers and plain yoghurt).

#### 3.4.4 Taste

Compared to control samples, all short set yoghurt samples formulated with CMC had lower taste scores and increased concentrations (from 0.5% to 1.0%). The decrease in the taste sensory scores in the CMC‐containing yoghurt samples was only found to be significant (*p* < .05) when the fermentation time was 3 hr.

There was an improvement in the taste sensory scores for all samples formulated with corn starch as fermentation progressed (3–5 hr). All samples containing corn starch had increased taste scores as the concentrations changed from 0.5% to 1.0%. There was an improvement in taste sensory scores observed in all samples formulated with gum acacia, which was significant (*p* < .05) with the increase in the fermentation time (3–5 hr). Samples containing 1.0% gum acacia (7.10 ± 0.55) were liked moderately. In comparison, gum acacia containing samples at 0.5% level (4.60 ± 1.47) while the fermentation lasted for 3 hr was found to have the least taste sensory scores. Further addition of gum acacia could increase dryness resulting in an adverse effect on the texture and taste of the samples (Pecivova et al., 2013).

#### 3.4.5 Overall acceptability

The control yoghurt samples (plain) had higher sensory scores for overall acceptability compared to the samples containing CMC, as concentrations increased from 0.5% to 1.0%. Compared to the control (short set yoghurt without stabilizer), there was an increase in the sensory scores for overall acceptability in yoghurt samples formulated with corn starch as concentrations increased. Fermentation was found to influence the sensory scores of samples in which corn starch was added because of higher values obtained as fermentation progressed (from 3 to 5 hr), but this was not significant (*p *> .05). The highest sensory scores or overall acceptability was found in samples formulated with corn starch at a 1.0% level. Samples in which gum acacia was incorporated showed a similar trend as obtained in samples containing corn starch. The effect of improvement in the overall acceptability scores obtained in gum acacia containing samples was significant (*p* < .05) as fermentation progressed, unlike those samples containing corn starch.

The interaction effect between stabilizers and concentrations on the sensory parameters showed that the differences between the stabilizers were different at different concentrations or that the differences between stabilizers on the color, taste, flavor, mouthfeel, and overall acceptability of short set yoghurt were magnified at higher concentrations.

## 4 CONCLUSION

The results obtained in this work indicated that the addition of stabilizers and the use of different fermentation times improved the proximate, physicochemical, microbial, and sensory properties of short set yoghurts. It was also observed that 1.0% concentration of CMC and corn starch was optimal for short set yoghurt samples fermented at 40–43℃ beyond which their usage becomes wastage when compared to gum acacia which appeared to require a higher concentration of more than 1.0% in order to equilibrate with 0.5%–1.0% CMC. Due to its significant impact on the desirable qualities—total solids, moisture, overall acceptability, and good keeping quality on the produced yoghurt samples compared to other stabilizers (Gum acacia and CMC), the use of corn starch is therefore recommended for the production of short set yoghurts. However, further improvement may be carried out on the sensory attributes and the acidity of short set yoghurt. Also, there is a need to improve gum acacia usage by combining it with other stabilizers to improve its rheological properties since it produces good textural properties in dairy products.

## CONFLICT OF INTEREST

The authors do not declare any conflict of interest before, during or after this study.

## AUTHOR CONTRIBUTION

**Chinazom Martina Eze:** Conceptualization (equal); Data curation (equal); Investigation (equal); Methodology (equal); Project administration (equal); Resources (equal); Writing‐original draft (equal); Writing‐review & editing (equal). **Kehinde Oludayo Aremu:** Conceptualization (equal); Data curation (equal); Formal analysis (equal); Investigation (equal); Methodology (equal); Writing‐original draft (equal); Writing‐review & editing (equal). **Emmanuel Oladeji Alamu:** Conceptualization (equal); Data curation (equal); Investigation (equal); Resources (equal); Validation (equal); Visualization (equal); Writing‐review & editing (equal). **Thomas Muoneme Okonkwo:** Conceptualization (equal); Data curation (equal); Funding acquisition (equal); Project administration (equal); Validation (equal); Visualization (equal); Writing‐review & editing (equal).

## Data Availability

The data will be made available at the request of the corresponding authors.
